# Supplementary material for: Effects of subclinical depression on prefrontal–striatal model-based and model-free learning
Source: PLoS Comput Biol. 2021 May 14;17(5):e1009003. doi: 10.1371/journal.pcbi.1009003 (PMC8153417; doi:10.1371/journal.pcbi.1009003)
Supplement: S1 File — (DOCX) [file pcbi.1009003.s001.docx]

**S1 File. Supplementary information:**

**Effects of subclinical depression on prefrontal–striatal model-based and model-free learning**

Suyeon Heo^1,2^, Yoondo Sung^1^ and Sang Wan Lee*^1,2,3,4,5^

^1^ Department of Bio and Brain Engineering, Korea Advanced Institute of Science Technology (KAIST), Daejeon 34141, Republic of Korea
^2^ Brain and Cognitive Engineering Program, Korea Advanced Institute of Science Technology (KAIST), Daejeon 34141, Republic of Korea
^3^ KAIST Institute for Health Science Technology, Korea Advanced Institute of Science Technology (KAIST), Daejeon 34141, Republic of Korea
^4^ KAIST Institute for Artificial Intelligence, Korea Advanced Institute of Science Technology (KAIST), Daejeon 34141, Republic of Korea

^5^ KAIST Center for Neuroscience-inspired AI, Korea Advanced Institute of Science and Technology (KAIST), Daejeon 34141, Republic of Korea


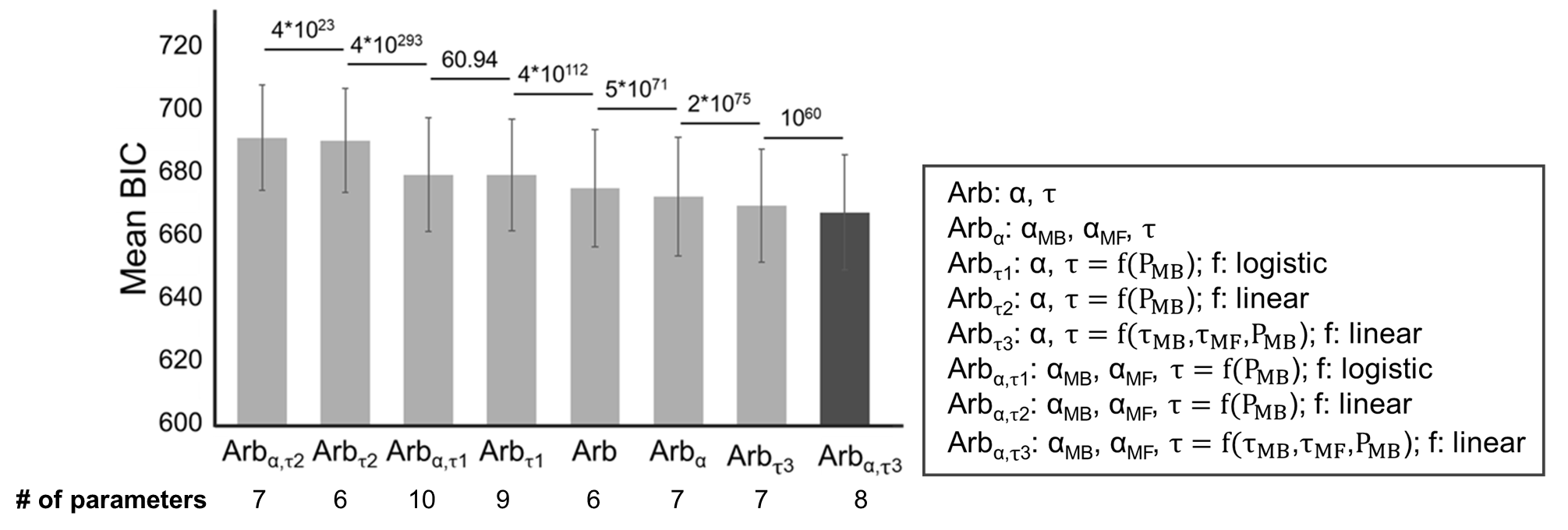


**Fig A. Model comparison**

To select the model that best describes a human behavioral data, we used an average Bayesian information criteria (BIC) across subjects. Arb refers to the original model (Lee et al., 2014). Arb_α_ modifies the Arb model, by considering the separate learning parameter between model-based and model-free system. Arb_α,τ_ develops Arb_α_, with further consideration of the dynamic exploitation parameter which is the function of how the agent is biased to each system. Three types of equations explaining the dynamic exploitation parameter are used: logistic, linear, and weighted linear.

[Arb_α,τ1_] Logistic function: $\tau=c_{1}+ \frac{c_{2} -c_{1}}{1+e^{(-c_{3}*\left( P_{\mathrm{MB}}-c_{4} \right))}}$, where c1: lower-bound, c2: upper-bound, c3: steepness of the curve, c4: midpoint of the function

[Arb_α,τ2_] Linear function: $\tau{=P}_{\mathrm{MB}}*c$

[Arb_α,τ3_] Weighted linear function: ${\tau{=P}_{\mathrm{MB}}*\tau}_{\mathrm{MB}}$ + (1-$P_{\mathrm{MB}})*\tau_{\mathrm{MF}}$

Bayes Factor was used to compare the superiority of each model. The numbers above the graph show the Bayes Factor for comparing two models. Error bar stands for the standard error.

**
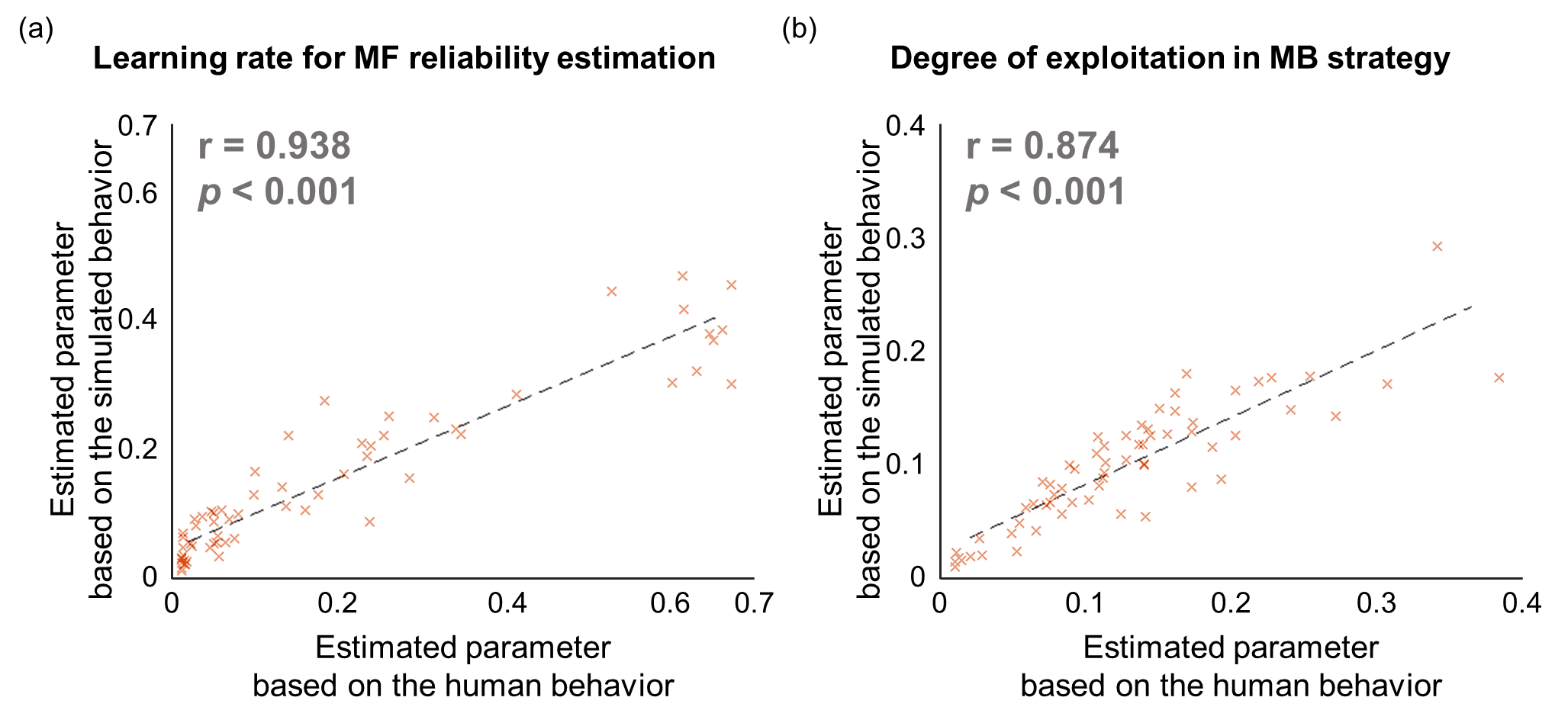
**

**Fig B. Parameter recovery of the two parameters related to the depressive symptoms.**

To assess whether the parameters of the best version of the model encodes the key characteristics of choice behavior, we ran a parameter recovery analysis, consisting of the following sequence of processes: Subjects’ original behavioral data → model fitting 1 (original parameters) → simulated behavioral data → model fitting 2 (recovered parameters). We compared the estimated parameter from subjects’ choice behavior (shown on the x-axis) and the estimated parameter from the simulated behavior (shown on the y-axis). Dots are the averaged simulated results for each subjects.

| x | y | z | Peak in region | Hemi | p (FWE) | z-score |
| --- | --- | --- | --- | --- | --- | --- |
| State Prediction Error (SPE) | | | | | | |
| 30 | 16 | -8 | Insula (sub-lobar part) | Right | 0.009 | 4.36^+^ |
| -28 | 16 | -10 | Insula (sub-lobar part) | Left | 0.001 | 4.62^+^ |
| -36 | 20 | -4 | Insula (Frontal-lobe part) | Left | 0.001 | 4.49^+^ |
| 50 | 8 | 24 | IPFC (lateral PFC) | Right | 0.000 | 4.24^+^ |
| -44 | 20 | 14 | IPFC | Left | 0.000 | 4.36^+^ |
| Reward Prediction Error (RPE) | | | | | | |
| -14 | 8 | -8 | Putamen | Left | 0.004 | 5.42* |
| 14 | 8 | -8 | Putamen | Right | 0.004 | 5.42* |
| 4 | 8 | -4 | Caudate | Right | 0.008 | 5.30* |
| 4 | 52 | 0 | Frontopolar PFC | Right | 0.000 | 4.76^+^ |
| -4 | 6 | -4 | Caudate | Left | 0.000 | 4.50^+^ |
| -14 | 28 | 58 | SFG (Superior Frontal Gyrus) | Left | 0.003 | 4.34^+^ |
| 2 | -38 | 40 | Posterior Cingulate Gyrus | Right | 0.031 | 3.77^+^ |
| Max Reliability (max(Rel_MB_, Rel_MF_)) | | | | | | |
| -52 | 26 | 16 | ilPFC (inferior lateral PFC) | Left | 0.001 | 4.48^+^ |
| 4 | 32 | 50 | FPC (Frontopolar PFC) | Right | 0.000 | 4.18^+^ |
| 42 | 32 | -8 | ilPFC | Right | 0.026 | 3.67^1^ |

**Table A. Neural representations of model-based and model-free prediction errors and arbitration signals**

*: threshold p<0.05 FWE corrected at the peak-level

+: survives whole-brain correction for multiple comparison at the cluster level

(extent>100 voxels, height threshold t=3.5)

1: survives small-volume corrected within a 10 mm sphere at [48, 35, -2] (Lee et al., 2014)

| x | y | z | Peak in region | Hemi | p (FWE) | z-score |
| --- | --- | --- | --- | --- | --- | --- |
| Chosen value of the model-based system only (Q_MB_) | | | | | | |
| 40 | -18 | 52 | Precentral Gyrus | Right | 0.001 | 3.92^+^ |
| Chosen value of the model-free system only (Q_MF_) | | | | | | |
| -8 | 20 | 32 | dACC (dorsal ACC) | Left | 0.000 | 4.40^+^ |
| -12 | 16 | 66 | SMA (Supplementary Motor Area) | Left | 0.044 | 4.34^+^ |
| -26 | 4 | 54 | Premotor Cortex | Left | 0.006 | 4.30^+^ |
| -34 | 30 | 26 | dlPFC (dorsolateral PFC) | Left | 0.000 | 4.22^+^ |
| -44 | -34 | 36 | IPL (Inferior Parietal Lobule) | Left | 0.011 | 4.12^+^ |
| 24 | 8 | 66 | Premotor Cortex | Right | 0.041 | 4.02^+^ |
| 48 | -56 | 26 | Supramarginal Gyrus | Right | 0.028 | 3.86^+^ |
| Value difference of the arbitration system – positive correlation (chosen-unchosen) | | | | | | |
| -36 | -24 | 48 | Postcentral Gyrus | Left | 0.000 | 6.32* |
| -50 | -58 | 24 | STG  (Superior Temporal Gyrus) | Left | 0.004 | 4.08^+^ |
| 4 | 40 | -6 | vm/omPFC  (ventral medial/orbital&medial PFC) | Right | 0.001 | 3.89^+^ |
| Value difference of the arbitration system – negative correlation (unchosen-chosen) | | | | | | |
| 0 | 28 | 50 | SMA |  | 0.024 | 5.06* |
| 30 | 30 | 0 | Insula | Right | 0.055 | 4.86* |
| -40 | 14 | -6 | Insula | Left | 0.000 | 4.73^+^ |
| 12 | -14 | 8 | Thalamus | Right | 0.002 | 4.62^+^ |
| 58 | 18 | 20 | ilPFC | Right | 0.001 | 4.34^+^ |

**Table B. Neural representations of value signals**

*: threshold p<0.05 FWE corrected at the peak-level

+: survives whole-brain correction for multiple comparison at the cluster level

(extent>100 voxels, height threshold t=3.5)

| x | y | z | Peak in region | Hemi | p (FWE) | z-score |
| --- | --- | --- | --- | --- | --- | --- |
| Probability of chosen action – positive correlation (P_action chosen_) | | | | | | |
| -36 | -50 | 48 | IPL | Left | 0.000 | 6.33* |
| 28 | 24 | 6 | Insula | Right | 0.003 | 5.47* |
| -30 | 20 | 4 | Insula | Left | 0.012 | 5.23* |
| 38 | -44 | 56 | IPL | Right | 0.034 | 4.99* |
| -44 | 30 | 26 | MFG (Middle Frontal Gyrus) | Left | 0.000 | 4.84^+^ |
| 10 | 0 | -2 | Globus Pallidus | Right | 0.013 | 4.58^+^ |
| 12 | 6 | 0 | Globus Pallidus | Right | 0.001 | 4.41^+^ |
| -50 | 8 | 24 | Inferior Frontal Gyrus | Left | 0.000 | 4.37^+^ |
| 0 | 26 | 44 | FPC |  | 0.000 | 4.24^+^ |
| 12 | 16 | 46 | SMA | Right | 0.000 | 4.00^+^ |
| 10 | -14 | 8 | Thalamus | Right | 0.011 | 3.98^+^ |
| Probability of chosen action – negative correlation (P_action chosen_) | | | | | | |
| -2 | 32 | -14 | Orbitofrontal Cortex | Left | 0.000 | 5.82* |
| 8 | 40 | -8 | Orbitofrontal Cortex | Right | 0.045 | 4.92* |
| 58 | -58 | 22 | STG | Right | 0.016 | 5.17* |
| -60 | -20 | -10 | MTG (Middle Temporal Gyrus) | Left | 0.017 | 5.16* |
| -48 | -56 | 24 | Supramarginal Gyrus | Left | 0.033 | 5.00* |
| 64 | -12 | -14 | MTG | Right | 0.008 | 4.63^+^ |
| 4 | 54 | 32 | mPFC (medial PFC) | Right | 0.012 | 4.44^+^ |
| 60 | -42 | 0 | MTG | Right | 0.013 | 4.33^+^ |
| -10 | 50 | 46 | SFG | Left | 0.000 | 4.07^+^ |

**Table C. Neural representations of valuation-action translation signals**

*: threshold p<0.05 FWE corrected at the peak-level

+: survives whole-brain correction for multiple comparison at the cluster level

(extent>100 voxels, height threshold t=3.5)

|  |  | **Mask from Lee et al. (2014)** | | | **Mask from current GLM analysis** | | |
| --- | --- | --- | --- | --- | --- | --- | --- |
| **ROI** | **Signal** | **Prediction performance (standard error)** | | | | | |
|  |  | **Healthy group** | **Subclinical depression group** | **P-value** | **Healthy group** | **Subclinical depression group** | **P-value** |
| FPC | Reliability of MB system | 65.9 (8.8) | 64.6 (5.8) | 0.650 (F_1,26_=0.21) | 64.1 (6.9) | 67.2 (10.3) | 0.352  (F_1,26_=0.90) |
|  | Reliability of MF system | 60.6 (3.6) | 63.5 (3.3) | 0.039*  (F_1,26_=4.72) | 60.1 (3.6) | 63.5 (4.5) | 0.036*  (F_1,26_=4.89) |
|  | Max reliability | 61.9 (5.6) | 63.3 (3.6) | 0.456  (F_1,26_=0.57) | 61.7 (3.7) | 63.5 (5.0) | 0.307  (F_1,26_=1.09) |
| Left ilPFC | Reliability of MB system | 68.8 (6.7) | 68.0 (5.5) | 0.713  (F_1,26_=0.14) | 62.6 (6.9) | 62.4 (4.9) | 0.925  (F_1,26_=0.01) |
|  | Reliability of MF system | 62.9 (4.5) | 66.8 (5.0) | 0.044*  (F_1,26_=4.49) | 60.0 (3.9) | 62.6 (5.0) | 0.042*  (F_1,26_=4.57) |
|  | Max reliability | 67.1 (6.2) | 65.6 (4.2) | 0.493  (F_1,26_=0.48) | 61.0 (3.8) | 62.4 (4.6) | 0.393  (F_1,26_=0.76) |
| Right ilPFC | Reliability of MB system | 64.3 (5.3) | 65.4 (5.5) | 0.605  (F_1,26_=0.27) | 63.3 (8.1) | 64.4 (7.2) | 0.731  (F_1,26_=0.12) |
|  | Reliability of MF system | 59.7 (4.0) | 64.1 (5.3) | 0.019*  (F_1,26_=6.28) | 60.4 (3.1) | 63.4 (4.2) | 0.047*  (F_1,26_=4.33) |
|  | Max reliability | 63.1 (5.5) | 63.7 (4.0) | 0.726  (F_1,26_=0.13) | 61.7 (3.7) | 63.5 (5.0) | 0.307  (F_1,26_=1.09) |

**Table D. Arbitration information prediction performance in bilateral ilPFC and FPC**

MVPA prediction performance for normal and subclinical depression group is mentioned in the table. Masks are defined in two ways. Mask from Lee et al. (2014) indicates that the ROI definition was based on the GLM results from Lee et al. (2014) data set. Mask from current GLM analysis indicates that the mask is defined by the data set used in this study. The clusters whose voxel activity survives at whole-brain correction with threshold p<0.001 (uncorrected) are defined as a mask. Asterisk (*) indicates significant inter-group difference at the 0.05 level.
