# Supplementary material for: Effects of subclinical depression on prefrontal–striatal model-based and model-free learning
Source: PLoS Comput Biol. 2021 May 14;17(5):e1009003. doi: 10.1371/journal.pcbi.1009003 (PMC8153417; doi:10.1371/journal.pcbi.1009003)
Supplement: Attachment — Submitted filename: Response to reviewers.docx [file pcbi.1009003.s003.docx]

**Editor’s comment**

As with all papers reviewed by the journal, your manuscript was reviewed by members of the editorial board and by three independent reviewers. In light of the reviews, we would like to invite the resubmission of a significantly-revised version that takes into account the reviewers' comments.

As the authors can see from the reports below, all referees appreciate the aim of the study and the use of the MF/MB-arbitration task in this dimensional sample. The referees' main areas of concern are (i) a lack of motivating certain analyses, (ii) the multitude of different analyses performed without clearly motivating them or correcting for multiple comparisons, (iii) a lack of details on methods and analyses. We expect the authors to address all concerns raised by the referees.

>> We are happy to see that all of the reviewers appreciate the novelty and significance of our results. We have done our best to fully resolve the issues raised by the reviewers. In the below, we have provided point-by-point responses to the reviewers’ concerns.

We greatly appreciate the reviewer’s very insightful and constructive comments, which have made it possible for us to significantly improve our paper.

**Reviewer’s comments**

**Reviewer #1**: The authors present an well-written study investigating model-based, model-free and the arbitration between the two modes of decision making in sub-clinical depression. Specifically, they found that depression was linked to sub-optimal choices in the two-stage Markov decision task. Subsequently, they expanded a previous model of arbitration control between model-based and model-free to include separate learning rates for model-based and model-free (to allow for sub-optimality in learning values) and defining an exploitation sensitivity parameter (to allow for sub-optimality in converting learned values into choice behavior). In addition to replicating basic model-based and model-free neural correlates, the authors also examined the relationship of these representations with individual differences in depression score. There was evidence that depression was negatively linked to SPE and RPE representations, and that prediction performance of model-free reliability was higher in more depressive participants, suggesting that depressive participants' arbitration of action control are over-sensitive to model-free updates. Finally, exploitation for model-based RL was also negatively associated with depression. Together, these findings highlight various reinforcement learning deficits in depression in model-free, model-based as well as in the arbitration of the two systems.

The Abstract and Summary were concise and accurate, and the main text was easily readable. I am generally impressed by the rigorous report and data presented. The analyses and discussion were also very comprehensive. However, there are several points below which I hope the authors can address:

- I find the description of the task in both Fig 1 and in the Methods slightly unclear, which may have contributed to my confusion about the task paradigm. The authors state that there are four main sessions with 80 trials: (1) Is this 80 trials per session or 80 trials overall? (2) Where the transition probabilities are manifested is also unclear. There are two state-transition probabilities (0.5, 0.5) and (0.9, 0.1), which are later mentioned to be interleaved with the uncertainty condition in a factorial design. (3) Does this mean that for a trial with (0.9, 0.1) probability, the 0.9 probability applies to two transitions, for one of both the left and right S1 choice each, and vice-versa for the 0.1 probability? (4) Similarly for the stage-two to outcome transitions, do these also follow the (0.5, 0.5) and (0.9, 0.1) probabilities of the earlier the state transition? This should be depicted more explicitly Figure 1a as well.

>> (Reply 1-1)

Re: (1). There are four sessions in total, each of which consists of 80 trials on average.

Re: (2). The transition probabilities are not shown to the subjects. Subjects need to estimate these values by experiencing state-transitions. This is an implicit manipulation intended to encourage engagement of model-based learning strategy. To fully clarify this, we have updated explanations about our task in the main text.

*“Participants were not informed about the state-transition probabilities in the task, but they were told that these might change during the task.”*

Re: (3) and (4). Yes, the state-transition probability applies to both transitions (both the first and the second stage). To clarify this, we have updated Figure 1.

(Previous Figure 1)

(Updated Figure 1)

- I would suggest that the authors include a signpost before the neural results that the fMRI data was examined with a subset (n=28) of the total behavioral group in the main text.

>> (Reply 1-2) Thanks for the suggestion. We have added this note to the main text.

- Was there any power analysis conducted for this study/How was the sample size determined?

>> (Reply 1-3) Although the power analysis provides a general guideline for the sample size, it cannot fully accommodate the context of experiments, such as similarities and interdependence of the individuals within a group, which may affect variance or data distributions per se. Instead, we followed the convention of other related works, whose average sample size varies between 36 and 133 (Beevers et al. 2013; Blanco et al. 2013; Kunisato et al. 2012; Miyagi, Miyatani, and Nakao 2017; Ogishima et al. 2020). To preclude a potential undersample issue, we have applied consistent and stringent criteria to our statistical tests (multiple corrections, p<0.05). Indeed, most of our main results have a significance level *p*<0.001.

- The authors mention that the learning of state-action value is unrelated to depression, however I struggle to find these results in the Results section.

>> (Reply 1-4) We apologize for causing the misunderstanding about the implication of our results. The corresponding sentence in the first paragraph of the discussion has a typographical error; “learning of the state-action value” should be corrected to “the state-action value” (Q value). Our findings indicate that mild depression significantly influences the way the action is updated (state and reward prediction error - Fig 6, arbitration control - Fig 7), rather than the action value itself. We have corrected this sentence as follows:

*“On the other hand, the expected state-action value itself is seemingly not influenced by the subclinical depression, which is consistent with the previous finding that the expected value is intact in MDD [45].”*

- It was highlighted that these RL deficits observed here are likely attributable to depressive, rather than anxiety, symptoms, which is a concern due to their high co-morbidity. Has there been specific studies of anxiety showing these similar deficits in RL, and did those studies control for depression?

>> (Reply 1-5) We appreciate the reviewer’s very insightful comment. One recent study using a probabilistic learning task showed that people with anxiety disorder appear to have a limited ability to learn and lower perceived reinforcement sensitivity after controlling for depression symptoms (LaFreniere and Newman 2018). A combination of the results from this study and our study would suggest potential factors that can dissociate depression from anxiety, so in the future, it would be interesting to use a RL task to better characterize the features of people exhibiting comorbidity. However, in this study, we realized that insufficient data presentation (we accumulated the anxiety score from some of the participants, n=51) and preliminary analysis on anxiety symptoms would unintentionally lead to confusion for the readers, so we decide to omit this argument from the discussion.

- Could the authors elaborate on this statement on pg 17, line 4-6. "First, simply evaluating the two separate hypotheses contradicts the prevailing view that the brain circuitry guiding goal-directed and habitual behavior interact with each other."? I don't understand how current evidence goes against the view.

>> (Reply 1-6) Sorry for the confusion. In this sentence, we intended to say that because both goal-directed (model-based) and habitual (model-free) system guide learning in the brain, if the assessment of the depression effect on learning is confined to one particular type of learning (either model-based or model-free learning), then we would not be able to fully evaluate the effect. To clarify this issue, we have revised the sentence.

*“First, the fact that both the model-based and model-free learning guide value-based decision making it necessary to incorporate the interaction between the two types of learning into the computational model.”*

- The participant group was split into two groups for the MVPA analysis, a "depressive" group and a "control" group. This classification is sudden given that the participants are not clinically diagnosed. In the methods it was mentioned that these groups are formed based on the clinical cut-off with the CES-D - this should be also referred earlier in the results. Another point is that there seems to be quite a high number (nearly half?) who cross the threshold for a depression diagnosis with this metric, especially from a recruitment that explicitly excluded psychiatric diagnoses. Is this common/expected in the sample recruited?

>> (Reply 1-7) Following the reviewer’s constructive suggestion, we have moved the corresponding sentence to the earlier part of the main text.

(Before Fig 4)

*To test the effect of depression on these behavior patterns, we split the subjects into “healthy group” (n=15) and “subclinical depression group” (n=13), based on the standard cutoff criterion of the CES-D score, 16 [42,43]. Note that this criterion was suggested to identify individuals at risk for clinical depression, not for diagnosing a depressive disorder.”*

(Before Fig 7)

*“To formally test the first hypothesis, we ran an MVPA for the bilateral ilPFC and FPC. … and compared the prediction performance of the control and subclinical depression groups (the same groups used in Fig 4).”*

Re: relatively high number of subjects above the depression threshold. As the reviewer pointed out, it is less likely that there are a large number of subjects with a high CES-D score. To preclude this data imbalance issue, after the initial screening with CES-D, subjects below the threshold were randomly selected for the fMRI study, whereas all the subjects above the threshold were fMRI-scanned. This strategy allowed us to obtain high individual variability in terms of the CES-D score while maintaining the balance between the low and high CES-D group.

Minor comments:

- Is age/gender a co-variate in any of these analyses?

>> (Reply 1-8) The age and gender are not treated as co-variates in our statistical analysis. Co-variate is controlled when the certain variable is correlated with both the dependent and independent variables; however, we did not find any correlation between age, gender, and depression score (i.e., CES-D score).

- Pg 9, line 2, P(MF) is not defined (1 - P(MB))?

>> (Reply 1-9) The basic assumption our computational model has is that the model-based and the model-free system compete to make optimal decision making. Therefore, the summation of the probability of choosing model-based and the model-free system is 1, indicating the P(MF) is equal to (1-P(MB)).

- Table E: GAD score, in the legend it says n = 23, but in the table it says n = 51?

>> (Reply 1-10) We have changed the legend to avoid the confusion. We gathered GAD scores from a total of 51 participants, 28 of whom participated in the fMRI test, and the remaining 23 participated in the behavioral test.

- Table D results could be represented in a landscape format, to compare Lee et al vs the current results side by side.

>> (Reply 1-11) We have relocated the format of the table.

**Reviewer #2**: The authors use a multistage choice task, fMRI, and computational modeling to examine how mild depression influence participants’ mediation between model-based and model free decision-making. The authors show that depression severity is negatively correlated with depressive severity, such that those individuals with lower levels of depression exhibit better performance (i.e., higher earnings through the course of the experiment). The authors fit the behavioral data using a modified reinforcement learning that accounts for model-based and model free choice, and the arbitration between the two; they use this modeling along with neuroimaging data to show that mild depression is associated with attenuated state and reward prediction error representation in the insula and caudate.

This is a potentially interesting paper that is ambitious in its integration of psychiatric classification of participants, behavioral modeling, and model-based fMRI. However, I am not convinced that the authors’ main findings are statistically valid and that the associated behavioral modeling and neuroimaing are combined in a scientifically coherent way. I’ve outlined my concerns below.

>> (Reply 2-1) We appreciate the reviewer’s insightful comments. We have done our best to fully address all the concerns, by providing point-by-point responses.

Behavior:

- Figure 1 shows correlations between accumulated reward, proportion of optimal choices, choice consistency, and depression score. However, the authors did not justify their use of the Center for Epidemiologic Studies Depression (CES-D) questionnaires. How and why was the scale chosen? Were other scales used? This scale has been shown to not align very well with DSM diagnosis of depression.

>> (Reply 2-2) This is an issue we took very seriously from the very early stage of our study.

For this, we consulted psychiatrists at the Department of medical science/psychiatry about making a shortlist of candidate depression scores, discussing advantages and disadvantages, conducting literature surveys, evaluating the consistency and reliability of the depression scores that our subjects submitted. We collected both CES-D and PHQ-9 data initially, and chose to use the CES-D score on the following basis.

The first rationale for using CES-D is that this score has been known to be reliable (Heo et al. 2018; Prescott et al. 1998). Our initial analyses suggested that statistical results from the PHQ-9 did not add up well (inconsistency in results from different model-based analyses), making us undermine the reliability of this measure. One reason for the insufficient reliability of the PHQ-9 might be ascribed to the fact that it is designed to be easily administered and scored; the length of PHQ-9 questionnaires is half that of CES-D.

Second, it is known that the CES-D is effective in capturing short-term symptoms (Zhang et al. 2015). This characteristic makes it easy for us to assess the effect of mild depression, for which development of symptoms may not be highly persistent.

Third, to maintain coherence with other related works, we conform to the convention of examining the effect of depression on decision making. For example, the following two studies used the CES-D score to examine the effect of depression symptoms on decision making. Note that they used a learning task with environmental uncertainty, which falls into the same category as our task.

(Kunisato et al. 2012) “Effects of depression on reward-based decision making and variability of action in probabilistic learning”

(Blanco et al. 2013) “The influence of depression symptoms on exploratory decision-making”

That said, we acknowledge that there have been ongoing debates about different characteristics of different depression measures, including a potential compatibility issue of the CES-D with DSM diagnosis of depression, and full investigation using a multitude of measures is left for future study.

To fully reflect the reviewer’s concern, we have added a new subsection (Rationale for using the CES-D score) to the discussion section.

- Why do the authors only relate model free behavior to depression measures (Fig 1)? It seems that if their computational models are truly sensitive to model based/model free arbitration their model parameters should be related to CES-D measures of depression. Was this not the case?

>> (Reply 2-3) Thank you very much for the insightful comment. The figure (Fig 2) that the reviewer mentioned is focused on the relationship between pure behavioral measures and the CES-D score. We did find and report multiple evidence showing that the model parameters of our computational model of arbitration are related to the CES-D measure, which are summarized in the following paragraph.

First, we showed the relationship between CES-D measure and the arbitration model’s parameter (Fig 7a; learning rate for the reliability of the model-free learning strategy). Second, we showed the relationship between CES-D measure and the neural activity of the brain region encoding the critical information of the arbitration model (Fig 7d; the left and right plot corresponds to the category and parametric effect, respectively). In addition, the relationship between CES-D measure and model-based learning is shown in Fig 8 (Fig 8a - parametric effect; Fig 8b - neural effect).

Note that all of this evidence coherently support our hypothesis about the effect of depression on model-based and model-free learning.

- Regarding behavioral modeling I’m curious about the extent of correlation between tau_mb and tau_mf and other RL model parameters. How behaviorally dissociable are these model parameters? This is important because it will impact the model-based neuroimaging that relies on these model parameters.

>> (Reply 2-4) Thanks for the insightful comment. Indeed, we ran a careful check to see if model parameters were fully dissociable.

First, note that the original experiment was carefully designed to separate out model-based from model-free learning. First, one can identify the different components of the Bellman equation associated with model-based (the red shaded box in the figure below) and model-free RL (the blue shaded box in the figure below), respectively. Then the two experimental parameters (goal and state-action-state transition probability) were defined based on these two components. As a result, manipulation of these experimental parameters directly serves to guide model-based and model-free learning.

Second, simulations were also performed with vanilla model-based and model-free algorithms in order to ensure that behavior and changes of the key latent state (reward and state prediction error) were fully dissociable. Note that prior to model-based neuroimaging analyses, we also ran GLM analyses for each subject’s data to check dependency between the time series of latent states of our computational models.

Third, we also ran a full correlation analysis after each model was individually fitted to each subject data. Shown below is the table of correlation between the free parameters of our computational model.

*: <0.05. Parameter 1: the threshold for classifying zero state prediction error; Parameter 2: the learning rate for the estimate of |RPE|; Parameter 3: the amplitude of a transition rate function (model-based → model-free); Parameter 4: the amplitude of a transition rate function (model-free → model-based).

Notably, among the parameters of model-based and model-free RL (corresponding to the 5th - 8th row and columns), there exists only one correlation between the tau of the model-based and the learning rate of model-free (rho=-0.2874, p=0.0224). That being said, the correlation level is not high (less than -0.3). Moreover, these two parameters are involved in a different level of computation (the former in value-action translation, the latter in value learning), precluding the possibility of impacting the model-based fMRI analysis.

Further remarks to prevent any possible misunderstanding about other existing correlations in the 1st - 4th row and columns: P1, P2, and P3 are the three parameters of the arbitration level that are functionally related, so their correlations are predictable. For example, a low threshold of zero SPE (P1) should influence the learning efficiency of its model-free counterpart (P2), and the increase in learning efficiency should facilitate transitioning to model-free learning.

- Overall, it seems strange that the authors’ computational model of arbitration does not covary with depression symptoms, as in the average behavior. This make me wonder if model-based/ model free arbitration is really influenced by depression, as the authors suggest. This is the main focus of the paper, so this point really needs to be flushed out.

>> (Reply 2-5) Thanks a lot for the constructive comments. To fully address this point, we ran additional behavioral analyses to confirm that our computational model of arbitration well accounts for behavior patterns associated with depression symptoms in all aspects: raw choice behavior, behavior guided by model-free learning (choice consistency (Lee, Shimojo, and O’Doherty 2014)) and behavior guided by model-based learning (choice optimality (Kim et al. 2019)). In the below, we provide a full clarification.

(i) Raw choice behavior

First, we examined whether the raw level behavioral patterns related to the depression symptom are explained by our computational model of arbitration (below figure). We used the proportion of actual choice of the agent, the behavioral measure used in [Lee, Neuron 2014, Kim Nature Comm 2019]. Specifically, it is the proportion of the right action (R) as a function of choice probability, which is computed based on the value difference between the two choices (R and L). Shown on the left and right is the behavioral measure of actual human subjects’ data and model simulation data, respectively. To examine the effect of the depression symptom on this behavior, we subdivided each case into the healthy (normal) and depressive group (depressive); they are the same as in Fig 6 of our original manuscript (Lee et al. 2014). Note that the model simulation case replicated behavioral patterns associated with the depression symptom (both the main and interaction effects; refer to the statistical test results).

(ii) Choice behavior associated with model-free and model-based learning

Next, we examined whether the model-free and model-based behavioral patterns related to the depression symptom are explained by our computational model of arbitration (below figure).

- (Figure (b) below) To quantify the behavior associated with model-free learning, we computed choice consistency (Kim et al. 2019; Lee et al. 2014) (also used in Fig 2c). This measure is defined as the proportion of making the same choice as in previous trials. Shown on the left and right is the behavioral measure of actual human subjects’ data and model simulation data, respectively.
  We found that the significant correlation between choice consistency and the depression symptom (CES-D) in subjects’ data (left) is replicated in the model simulation data (right) (see the scatter plot). The patterns are also replicated when we tested the effect of the key task variables (goal and uncertainty) on choice consistency (refer to the error bar plot and the statistical test results; both the main and interaction effects were replicated).
- (Figure (c) below) To quantify the behavior associated with model-based learning, we computed choice optimality*(Kim et al. 2019). This measure quantifies the extent to which a subject’s choice reflects an optimal policy; the optimal policy assumes that the precise uncertainty and goal information is immediately reflected in the model’s value. Shown on the left and right is the behavioral measure of actual human subjects’ data and model simulation data, respectively.
  We found that the significant correlation between choice optimality and the depression symptom (CES-D) in subjects’ data (left) is replicated in the model simulation data (right) (see the scatter plot). The patterns are also replicated when we tested the effect of the key task variables on choice optimality (refer to the error bar plot and the statistical test results; all the effects were successfully replicated, except for the uncertainty effect).

To fully reflect the reviewer’s comments, we have included these results as Fig 4 in the revised version of our manuscript (“Fig 4. Computational model of arbitration explains subjects’ behavioral patterns associated with depression symptom”).

*Additional note on choice optimality measure. In the revised version of the manuscript, we have replaced the proportion of optimal choices with the choice optimality [Kim et al. 2019]. This change has been made to Fig 2 and Fig 3. The old measure was defined as the ratio of the number of optimal choices to the number of total choices. Note that our new results are consistent with the previous results.

| Original figures | Updated figures |
| --- | --- |
| Fig 2b   | Fig 2b   |
| Fig 3c middle   | Fig 3c middle   |

Imaging

- It is great that the authors replicate the neuroimaging main effects from their 2014 paper. These correlations appear to be very strong.

>> (Reply 2-6) We are glad to see that the reviewer appreciates the importance of replicating the main effects reported in the previous work. These results indicate that the neural effect is very strong and that our computational model captures the essence of behavior associated with goal-directed and habitual learning. We believe that this helps to improve the credibility of the effect of mild depression on goal-directed and habitual control.

- The correlations between depression scores and neural effects (figure 5) seem to be very weak and not well motivated. The authors obtain strong bi-lateral insula and dlPFC activity in the main effect, but focus on left insula in the correlation with depression. In the RPE contrast they get strong vmPFC and putamen activity but individually look at left and right putamen correlations with depression score. More needs to be done to justify these ROI analyses.

>> (Reply 2-7) Thanks for offering valuable guidance on clarification of the ROI analyses. To prevent any bias/assumptions from affecting or imposing constraints on our analysis, we compiled the list of ROIs purely based on our model-based fMRI analysis (Fig 5). Because our computational models were fitted to subjects’ choice behavior data, as opposed to depression-specific data, we do not expect that all of the main effects from the model-based fMRI analysis are necessarily correlated with the depression score.

Accordingly, our ROI analyses employed an explorative approach. We considered all of the brain regions we identified from the model-based fMRI analysis, and reported the neural effects that passed the statistical significance test (Fig 6). This means that we tested insular bilaterally, but we did not find the effect in the right insula. This might be related to a few open questions, such as depression-specific lateralization of brain functions or biological characteristics of mild and situational depression that may not be clinically diagnosed (Bruder et al. 2016; Hecht 2010; Heller, Nitschke, and Miller 1998; Jiang et al. 2019; Li, Xu, and Lu 2018; Perez 2014).

We should note that given the fact that the severity of depression in our subjects is modest, it is very challenging to find the behavioral and neural effects. We think it is worthwhile to report these results of Fig 6, along with other neural effects (Fig 7 and Fig 8) in the main text, as they merit further investigation.

To fully clear up this issue, we have provided a justification of our ROI analyses in the Methods section and have added to the discussion section the above discussions about the results of Fig 5.

- I would also be interested to know if depression score correlates with Q-value or Pchosen. This is important information. What brain activity best describes the depression scores? This isn’t presented in the paper, so it is hard to understand the strength of neural results in the context of depression.

>> (Reply 2-8) Thanks for raising an interesting point. Yes, we agree with the reviewer that it would be interesting to know the correlation between depression score and valuation, including value (Q-value) and action-value conversion (Pchosen). Though it is seemingly a simple question, the underlying issues are more complicated than it sounds.

First, each subject experiences different episodes of events caused by different choices and uncertainty of the environment. This makes it hard to directly relate subject experience to the depression score (e.g., average Q-value or the average number of choices). Second, these are a few potential confounding factors contributing to choices, such as individual performance, choice bias, etc., which may or may not be associated with depression.

Our study avoids the above issues by focusing on model-based/parametric analyses, aiming to investigate the depression effect on the “process” guiding choices. Specifically, our computational model of arbitration control, in which its parameters were individually fitted to subjects’ data, enables us to separate out the characteristics of choice behavior from those associated with various individual variability, ranging from learning performance and choice bias to the strategy bias towards model-free/model-based learning. Model fitting by itself can be viewed as the process of factoring out individual biases from the data.

Another benefit of our computational approach is that the model helps us separate out subject experience from episodes of events contingent on environmental dynamics (e.g., reward, state-transition uncertainty, goal changes). In that regard, one key variable of our computational model is the prediction error signal, representing the discrepancy between the agent’s expectation about an outcome and the actual outcome given by the environment. Intriguingly, we found the effect of depression on reward prediction error in bilateral caudate. This finding is consistent with the results from previous studies (Dombrovski et al. 2013; Gradin et al. 2011; Kumar et al. 2008, 2018; Rothkirch et al. 2017; Ubl et al. 2015). Note that our model-based neural analyses also yield new results showing the relationship between the depression score and state prediction error, arbitration control signal (reliability of the current learning strategy), and Pchosen (Fig 6, 7, 8).

To fully reflect the reviewer’s point, we have added the above points to the discussion section.

**Reviewer #3**: In their manuscript “Effects of mild depression on prefrontal–striatal model-based and model-free learning”, Heo et al. used behavioural testing, computational modelling and fMRI to examine MB and MF reinforcement learning in humans. The authors found that mild depressive symptoms are associated with reduced neural prediction error signals and impaired arbitration between MB and MF learning. Lastly, the authors also show that mild depression is related to reduced exploitation of rewarding options.

1) The authors mainly present correlational results between CES-D scores and behaviour/neural data. In the figures, however, and on p16 in the results section, the authors also refer to one group as “healthy”, and the other as “depressive”. I might have missed this, but I didn’t find a proper explanation for how these groups were defined in the methods section. Based on a particular cut-off score for symptoms? Based on a median split? Given that, as far as I understand, all subjects are supposed to be non-diagnosed with clinical depression by a psychiatrist or specific clinical standards, this seems highly problematic to me. Overall, I do not think that authors can say mild depression, given that subjects are not diagnosed with depression. It seems to me that some subjects seem to have subclinical depression, i.e., depressive symptoms but not necessarily meeting criteria for depressive disorder. Also, it is not appropriate to refer to subjects as having “early” depression. This study is cross-sectional, and we do not know anything about the trajectory of symptoms of the subjects. The authors need to be clearer about their procedure, and in turn, define their research questions appropriately.

>> (Reply 3-1) We apologize for not providing sufficient information about the subject groups in the main text, which caused misunderstandings. In the below section, we fully clear up issues raised by the reviewer.

Subject groups based on a clinical standard. In the original manuscript, we provided in the Method section a clear definition of classification of subjects into two groups. The classification is based on the standard cutoff criteria of the CES-D score (=16) (Comstock and Helsing 1976; Weissman et al. 1977). We conform to the convention of the Center for Epidemiological Studies-Depression provided by the American Psychological Association (https://www.apa.org/pi/about/publications/caregivers/practice-settings/assessment/tools/depression-scale, Lewinsohn et al. 1997), stating that the CES-D cutoff score (e.g., 16 or greater) aids in identifying individuals at risk for clinical depression, with good sensitivity and specificity and high internal consistency (Lewinsohn et al. 1997) and has been used successfully across wide age ranges (Lewinsohn et al. 1997).

Usage of the term “mild depression”. We fully agree with the reviewer that mild depression should not be confused with clinically diagnosed depression. According to Lewinsohn et al. (1997), subjects with the CES-D score above the cutoff score 16, which we used in this study, fall into the category of people with a “risk for clinical depression”. Though a few group studies used this criterion to investigate depression (Beevers et al. 2013; Blanco et al. 2013; Kunisato et al. 2012), in both the title and the manuscript, we have changed the term “mild depression” to “subclinical depression”, which refers to a condition in which a person has depressive symptoms but neither meet the standard criteria for a depressive disorder nor is clinically diagnosed as depression.

To fully reflect the reviewer’s concerns and also to avoid any misunderstanding about the subject group definition, we have included the details about the subject groups in the main text as well.

(Before Fig 4)

*To test the effect of depression on these behavior patterns, we split the subjects into “healthy group” (n=15) and “subclinical depression group” (n=13), based on the standard cutoff criterion of the CES-D score, 16 [42,43]. Note that this criterion was suggested to identify individuals at risk for clinical depression, not for diagnosing a depressive disorder.”*

(Before Fig 7)

*“To formally test the first hypothesis, we ran an MVPA for the bilateral ilPFC and FPC. … and compared the prediction performance of the control and subclinical depression groups (the same groups used in Fig 4).”*

2) The authors present three correlations of model-free behavioural data and depression scores (Fig. 2). What is the rational for presenting these correlations only? I am sure, there are many other behavioural model-free variables? Would be good to know whether the authors had specific hypotheses? Also, I am not sure if I understand the reasoning for using Pearson and Spearman. Are the depression scores normally distributed?

>> (Reply 3-2) We appreciate the reviewer making insightful comments about the rationale for our model-free behavioral analyses, which greatly helped us better establish a link between our hypothesis and the behavioral measures.

(i) Rationale for three model-free behavioral measures (reward, choice optimality and consistency)

Our model-free behavioral analysis aims to explore pure behavioral effects of subclinical depression on choice behavior. In doing so, we used distinctly different three behavioral measures, each of which is associated with overall learning performance, performance of model-based learning, and performance of model-free learning. Specifically, the first behavioral measure, accumulated reward (Fig 2a), is a conventional measure to assess behavioral performance. This helps us evaluate the relationship between overall learning performance and the CES-D score. Consistent with previous views, a negative relationship was found.

This result motivates us to dissect performance in terms of model-based and model-free learning. The second behavioral measure, the choice optimality* (Fig 2b), is a behavioral read-out for model-based control (Kim et al. 2019; Lee et al. 2014), whereas the third measure, choice consistency (Fig 2c), is a behavioral read-out for model-free control (Lee et al. 2014). Definitions and justifications of each measure are provided in the following two sections (ii) and (iii).

(ii) Choice optimality as a proxy for the model-based control effect on choice behavior

The choice optimality measure quantifies the extent to which participants on a given trial took objectively the best choice had they complete access to the task state-space, and a perfect ability to plan actions in that state-space. It is based on the choice of the ideal agent assumed to have full, immediate access to information of the environmental structure, including state-transition probability and goal changes. It is defined as the degree of match between subjects’ actual choices and an ideal agent’s choice corrected for the number of available options. To compute the degree of choice match between the subject and the ideal agent, for each condition, we calculated an average of normalized values (i.e., likelihood) of the ideal agent for the choice that a subject actually made on each trial. The choice optimality value would have a maximum/minimum value if a subject made the same/opposite choice as the ideal agent’s in all trials, regardless of complexity condition changes.

Owing to the fact that the ideal agent’s behavioral policy is not affected by the variability of such experimental variables, it serves as a good proxy for assessing the degree of participants’ engagement in model-based control. In principle, provided that the model-based agent has complete knowledge of the state-space and no cognitive constraints, it will always choose more optimally than a model-free agent. A separate computer simulation in one of our recent studies (Kim et al. 2019) has demonstrated that the choice optimality of the model-based agent is significantly higher than that of model-free agent (below figure).

*Additional note on choice optimality measure. In the revised version of the manuscript, we have replaced the proportion of optimal choices with the choice optimality [Kim et al. 2019]. This change has been made to Fig 2 and Fig 3. The old measure was defined as the ratio of the number of optimal choices to the number of total choices. Note that our new results are consistent with the previous results.

| Original figures | Updated figures |
| --- | --- |
| Fig 2b   | Fig 2b   |
| Fig 3c middle   | Fig 3c middle   |

(iii) Choice consistency as a proxy for the model-free control effect on choice behavior

Choice consistency, also known as choice repetition, is a conventional behavioral measure used to quantify insensitivity to changes in the environmental structure (one of the key characteristics of model-free RL), works well for conventional two-step task paradigms in which the environment is stable for a certain period (Daw et al. 2011; Miller, Botvinick, and Brody 2017). For example, when one outcome is associated with a relatively large amount of reward, a model-free agent is likely to repeat the same choice regardless of changes in environmental structures. It is known to account for habitual behavior. These characteristics make it easy to evaluate behavioral bias attributable to model-free control.

However, we are aware of its limitation in that it may not be suitable for highly dynamic task design which necessitates a mixed contribution of model-based and model-free control, so we used model-based analyses (Fig 3) to fully investigate separate contributions of model-free and model-based control on behavioral performance and its relationship with depression score (Fig 6, 7, and 8).

(iv) Other issues on correlation analyses

We used Pearson correlation because both depression score and behavioral data roughly followed normality condition (Depression score: Anderson Darling normality test, *p*=0.283); note that our statistical significance is not highly sensitive to the choice of tests. As our behavioral results corroborate a general view that depression might be associated with suboptimal choices, we try to remain cautious about reaching a conclusion solely based on this analysis. In the future, it would be interesting to apply our three behavioral measures to a large-scale clinical data.

To fully reflect the reviewer’s comment, we have provided the rationale for the behavioral measures of Fig 2 (see below). We have also added to Methods a new section “Model-free behavioral measures” which delineates the above points.

3) I am wondering whether the authors could relate the modelling results more to the model-free results they are showing in Fig. 2? As is, it appears subjects with higher depression scores are performing poorly in the task. Can the authors unpack that, potentially explain it with the results from the modelling, i.e., differences in learning? There is no relationship of results from model-free and model-based analyses mentioned as far as I can tell.

>> (Reply 3-3) We thank the reviewer for making a very constructive suggestion. The results from model-free (Fig 2) and model-based analyses (Fig 6, 7, and 8) are actually very coherent (as delineated in the following sections), we fully agree with the reviewer that this point merits in-depth discussion!

In the model-free analyses (Fig 2), we used three behavioral measures, each of which is intended to provide a rough estimation of overall learning performance, the degree of engagement of model-based learning and model-free learning, respectively (full details are provided in the Reply 3-2). We did find a negative relationship between these measures and the depression score, suggesting the possibility that subclinical depression might affect the performance of model-based and model-free learning.

(i) Model-based and model-free learning performance. The above results are fully consistent with the results of our model-based parametric GLM analyses (Fig 6). First, we found that the extent to which the insula encodes the state-prediction error signal, the key variable for model-based learning, is negatively correlated with the depression score (Fig 6a). This result corroborates the above behavioral finding (Fig 2b), showing that subclinical depression might have a negative impact on model-based learning. Second, we found that the extent to which the caudate encodes the reward-prediction error signal, the key variable for model-free learning, is negatively correlated with the depression score (Fig 6b). This result corroborates the above behavioral finding (Fig 2c), showing that subclinical depression might have a negative impact on model-free learning.

(ii) Arbitration performance. It appears that the decrease in the overall performance as a function of the depression score in our model-free analysis (Fig 2a) can be explained by the model-based MVPA analysis (Fig 7) meant to examine the performance of prefrontal arbitration control. Specifically, we found that the depression score is negatively correlated with the level of stability of the reliability estimation process (Fig 7a). In other words, the subclinical depression group showed less stable reliability estimation (Fig 7b), undermining the stability of the competition between model-based and model-free learning strategy (Fig 7c and 7d). This means that subjects with a lower depression score are more likely to use a suboptimal strategy that is less suitable for the current context (e.g., goal or state-transition uncertainty), leading to the impairment of overall learning performance (Fig 2).

(iii) Value-action conversion. Another potentially interesting factor that may influence the overall performance (Fig 2a) is value-action conversion, which is related to the ability to exploit viable options. We found that the degree of exploitation of the model-based learning strategy is negatively correlated with the depression score (Fig 8a). This suggests that the value information learned by the model-based strategy is less likely to be reflected in actual choices, essentially leading to the decrease in not only overall performance (Fig 2a and 2b) but also consistency of choices (Fig 2c).

Following the reviewer’s suggestion, in the revised version of the manuscript, we have added the above discussions to the discussion section.

4) Related to 3): subjects with higher depression scores perform worse. Thus, I am wondering whether the authors have collected any other cognitive scores for subjects to further unpack this result.

>> (Reply 3-4) We have not collected other cognitive scores as the current study focuses on investigating subclinical depression in the context of model-based and model-free reinforcement learning. That being said, a few studies examined the depression in terms of other cognitive functions that may advance our view (Chen et al. 2015; Huys, Daw, and Dayan 2015; Maddox et al. 2014; Markman and Miller 2006; Quelhas et al. 2008). For example, depressive patients tend to have less counterfactual thinking or show a deficit in reflective optimal learning. Since depression is known to be associated with complex traits of behavior, it would be interesting in the future to establish a link between various cognitive scores and the different layers of our computational model (e.g., model-free/model-based, arbitration, and valuation).

5) Related to 2): would be important to know what the relationship between the three task measures from Fig.2 is, e.g., how choice consistency is related to task performance, so as to understand the link between these correlations, and the behavioural performance in the task in general.

>> (Reply 3-5) Please refer to the Reply 3-3, in which we provide a full clarification of the three task measures of Fig 2.

6) In Fig. 3, the authors nicely explain the different models tested. However, for the understanding of the modelling section, it would be great if the authors could provide more detail in the methods, e.g., the fact that they use two distinct learning rates, αMB & αMF. I’d suggest the authors provide the equations for the different models in more detail.

>> (Reply 3-6) Thanks for the constructed comments. We have added a new paragraph explaining the model details to the Methods section.

7) Why are the authors specifically thresholding to a cluster size of 100 voxels (p29)?

>> (Reply 3-7) We used a cluster size of 100 voxels following the method from (Lee et al., 2014). Since our study modified the computational model from the previous work, the first work we should do is to test the replicability of the known neural findings to ensure the reliability of the new model. For this, we brought the same criteria from the previous study in reporting the neural results.

8) “For some areas reported from previous studies, we ran a 10 mm small-volume correction.” Given that the authors present a multitude of imaging results, which is not easy to follow when reading the manuscript, the above sentence is not appropriate. The authors need to be clear about i) which regions they looked at for which contrasts, ii) why they have chosen these regions, based on the canon of knowledge in the literature, iii) how many tests they ran for how many different regions (multiple comparison correction!), iv) why they have chosen 10mm (because for some, small brain regions, this seems quite a large threshold), v) how these regions were defined, using pre-defined anatomical masks or previous imaging studies as reference, and vi) whether they used a sphere/mask etc.? There is much more information needed here for the reader to understand this.

>> (Reply 3-8) We apologize for leading the confusion when applying the small-volume correction method without mentioning the logical basis. Similar to Reply 3-7, the main reason we used a small-volume correction is to fully reproduce the neural correlates from the previous work (Lee et al. 2014). However, the use of the small-volume correction is not directly related to the depression effects we are focusing on. From the reviewer’s comment, we recognized that the application of a 10 mm small-volume correction is not logically understandable to the reader and even makes the reader confusing, so we decided to remove most of the results from the main text. However, we left one area, ilPFC, an area known to engage in the metacognitive process (Baird et al. 2013; Lee et al. 2014; De Martino et al. 2013). We focused on this area to understand the disruption of the model-based and the model-free reliability signal, known to compete in the arbitration process, processing in subclinical depression. We chose a 10 mm criteria based on the previous work, which applies 10 mm sphere criteria in the lateral PFC region (Fujiwara et al. 2009). We referred to (Lee et al. 2014) to attain the standard region of the right ilPFC ([48, 35, -2]). Overall, we reported the right ilPFC region [42, 32, -8], area survives after a small-volume correction within a 10 mm sphere at [48, 35, -2]. Following the reviewer’s suggestion, we revised the small volume correction method in the method section.

9) The authors report that 65 subjects did the task, of which 28 were scanned. Did the fMRI subjects perform two sessions? Or only one (in the scanner)? Were the timings etc. identical between behavioural only and imaging?

>> (Reply 3-9) We apologize for not giving detailed information about the task. fMRI subjects and behavioral only subjects performed the identical task, except fMRI subjects conducted the whole task inside the fMRI scanner. They all performed 1 pre-training session and the 4 main sessions inside the scanner. The task time was also identical. Following the reviewer’s suggestion, we added the following sentence in the method section.

*“Both the behavior-only subjects and the fMRI subjects performed the tasks under the identical experimental setting except that the fMRI group conducted the experiment inside the scanner.”*

10) How long was the task (in minutes, volumes for fMRI)? What is the exact timing of the trials? There is a lot of methodological information missing as far as I am concerned.

>> (Reply 3-10) The pre-training session took 12 to 20 minutes, main session took 12 to 15 minutes/trial (fMRI volume: 280~340 scans/trial). In each trial, the state transition time was randomly determined from a uniform distribution between 1 to 4 seconds after making the action. The trial-to-trial interval was also drawn from the same distribution. The reward picture appeared for 2 seconds. Participants took approximately 80 minutes to perform the task. Following the reviewer’s suggestion, we have updated this information to the method section.

*“In each trial, state transition occurs between 1 to 4 seconds after making a choice; transition timing was sampled from a uniform distribution [1,4]. The intertrial interval was also drawn from the same distribution. The reward picture appeared for 2 seconds. The pre-training session was intended to provide subjects an opportunity to acquire a reasonable amount of knowledge about the state space. It took 12 to 20 minutes. The main session took 12 to 15 minutes per session. Participants took approximately 80 minutes to perform the task.”*

11) There is a lot of causality implied in the wording. That is, depression (which is critical, given as mentioned in 1), subjects are not even diagnosed with depression!) “disrupts” (abstract) or “driven by” (p16). “First, our neural results explain why…” (p21). I’d suggest the authors be more cautious here, because the find correlational relations between sub-clinical symptom data from questionnaires and task data, but it is not appropriate to infer causality from this data.

>> (Reply 3-11) Thanks for the advice. As the reviewer mentioned, our work reveals the relationship between depression score and the disruption of reinforcement learning, which is hard to view as a causality. We have changed the word implying causality to relations.

12) In the discussion section, the authors suddenly mention anxiety symptoms. This is not mentioned before, also not in the methods section. Thus, I am unclear what to make of this. Is there a relationship between anxiety and depressive symptoms in this group of subjects (which you’d expect)? This data needs to be presented to properly understand these results. Then, in the limitations section, the authors mention that there is a lack of other measures, such as anxiety. This is very confusing.

>> (Reply 3-12) We apologize for not providing sufficient explanation. Since previous studies report the comorbidity of anxiety and depression (Kessler et al. 1996; Sartorius et al. 1996), we wanted to confirm that our findings were not ascribed to the anxiety effect (in our data, we found a significant positive correlation between depression score and the anxiety score [r=0.776, *p*<0.001]). To reveal this, we gained the individual’s anxiety level (GAD-7 score) from some of the participants and analyzed the anxiety effects of reinforcement learning. However, we acknowledged that we gave an insufficient description of anxiety symptoms. This is still an open issue that merits full study, so to avoid any confusion, we have decided to omit this argument from the discussion.

13) What does “peak-level corrected” refer to in the fMRI results section? Is this p<0.05, FWE whole brain? This needs to be clearly stated at some point in the manuscript, e.g., in the methods.

>> (Reply 3-13) Peak-level corrected coordinates indicate the survival after the whole-brain correction for multiple correction at p<0.05 corrected level. To avoid confusion for the readers, we have changed the term as ‘p<0.05 FWE corrected’.

14) “It would be possible that this model can be used to predict the severity of mild depression as this model is fitted with individual behavioral data.” (p17). This seems an overly strong statement to me. The authors need to explain this, and why they think this is the case, given the data at hand.

>> (Reply 3-14) We meant that our model could be fitted to individual subject’s data with a different level of severity of depression, so assuming there exists enough individual variability, the mild depression can be projected onto model’s parameter space. If we had a new subject’s data to which we fit the model, we would be able to use the fitted parameter to map this onto the severity of depression. To respectively reflect the reviewer’s comment, we tone down the sentence so that this topic can be suggested as future works.

*“Future works can use this model to predict the severity of subclinical depression as this model is fitted with individual behavioral data.”*

15) “However, as other possibilities still remain to be explored, e.g., intact RPE representation in MDD [46]” (p19). May be worth discussing that Rutledge et al. found this in a non-learning task, which is different to the one the authors used here.

>> (Reply 3-15) Thanks for sharing an interesting work. We think that explaining the difference between the learning and non-learning case in terms of depression would be an interesting future direction. We have added a new sentence citing this work to suggest potential future work.

*“Another open question that merits further investigation is potential influence of depression on different types of cognitive processes. For example, there is commonalities and differences between our results with a learning task and Rutledge’s (45) work focused on a non-learning task structure. We believe that the exploring the difference between the learning and non-learning case in terms of depression would be an interesting future direction.”*

16) What does it mean that the two subjects that were excluded performed “below the chance-level” (p23)?

>> (Reply 3-16) The term ‘chance-level’ means the level of task performance of randomly behaving agents. As we mentioned in Reply 3-2, we defined an accumulated reward as the overall performance measure. We set the average accumulated reward of 10000 random simulations as the criterion for minimal behavioral performance. We excluded subjects whose performance is less than the chance-level because they were suspected that (1) they miss several responses during the task or (2) they intentionally fail to make an optimal choice, which leads to inaccurate behavioral modeling.

17) “pre-learning” and “pre-training” (p23), should this not be the same?

>> (Reply 3-17) Sorry for the confusion. We standardized the choice of the term as ‘pre-training’.

18) “p<0.001, uncorrelated” should be “uncorrected” (p30).

>> (Reply 3-18) We corrected the error. Thanks for pointing it out.

**References**

Baird, Benjamin, Jonathan Smallwood, Krzysztof J. Gorgolewski, and Daniel S. Margulies. 2013. “Medial and Lateral Networks in Anterior Prefrontal Cortex Support Metacognitive Ability for Memory and Perception.” *The Journal of Neuroscience : The Official Journal of the Society for Neuroscience* 33(42):16657–65.

Beevers, Christopher G., Darrell A. Worthy, Marissa A. Gorlick, Brittany Nix, Tanya Chotibut, and W. Todd Maddox. 2013. “Influence of Depression Symptoms on History-Independent Reward and Punishment Processing.” *Psychiatry Research* 207(1–2):53–60.

Blanco, Nathaniel J., A. Ross Otto, W. Todd Maddox, Christopher G. Beevers, and Bradley C. Love. 2013. “The Influence of Depression Symptoms on Exploratory Decision-Making.” *Cognition* 129(3):563–68.

Bruder, Gerard E., Jorge Alvarenga, Karen Abraham, Jamie Skipper, Virginia Warner, Daniel Voyer, Bradley S. Peterson, and Myrna M. Weissman. 2016. “Brain Laterality, Depression and Anxiety Disorders: New Findings for Emotional and Verbal Dichotic Listening in Individuals at Risk for Depression.” *Laterality* 21(4–6):525–48.

Chen, Chong, Taiki Takahashi, Shin Nakagawa, Takeshi Inoue, and Ichiro Kusumi. 2015. “Reinforcement Learning in Depression: A Review of Computational Research.” *Neuroscience and Biobehavioral Reviews* 55:247–67.

Comstock, G. W. and K. J. Helsing. 1976. “Symptoms of Depression in Two Communities.” *Psychological Medicine* 6(4):551–63.

Daw, Nathaniel D., Samuel J. Gershman, Ben Seymour, Peter Dayan, and Raymond J. Dolan. 2011. “Model-Based Influences on Humans’ Choices and Striatal Prediction Errors.” *Neuron* 69(6):1204–15.

Dombrovski, Alexandre Y., Katalin Szanto, Luke Clark, Charles F. Reynolds, and Greg J. Siegle. 2013. “Reward Signals, Attempted Suicide, and Impulsivity in Late-Life Depression.” *JAMA Psychiatry* 70(10):1.

Fujiwara, Juri, Philippe N. Tobler, Masato Taira, Toshio Iijima, and Ken-Ichiro Tsutsui. 2009. “A Parametric Relief Signal in Human Ventrolateral Prefrontal Cortex.” *NeuroImage* 44(3):1163–70.

Gradin, Victoria B., Poornima Kumar, Gordon Waiter, Trevor Ahearn, Catriona Stickle, Marteen Milders, Ian Reid, Jeremy Hall, and J. Douglas Steele. 2011. “Expected Value and Prediction Error Abnormalities in Depression and Schizophrenia.” *Brain : A Journal of Neurology* 134(Pt 6):1751–64.

Hecht, David. 2010. “Depression and the Hyperactive Right-Hemisphere.” *Neuroscience Research* 68:77–87.

Heller, Wendy, Jack B. Nitschke, and Gregory A. Miller. 1998. “Lateralization in Emotion and Emotional Disorders.” *Current Directions in Psychological Science* 7(1):26–32.

Heo, Eun-Hye, Kyeong-Sook Choi, Je-Chun Yu, and Ji-Ae Nam. 2018. “Validation of the Center for Epidemiological Studies Depression Scale among Korean Adolescents.” *Psychiatry Investigation* 15(2):124–32.

Huys, Quentin J. M., Nathaniel D. Daw, and Peter Dayan. 2015. “Depression: A Decision-Theoretic Analysis.” *Annual Review of Neuroscience* 38:1–23.

Jiang, Xueyan, Yuedi Shen, Jiashu Yao, Lei Zhang, Luoyi Xu, Rui Feng, Liqiang Cai, Jing Liu, Wei Chen, and Jinhui Wang. 2019. “Connectome Analysis of Functional and Structural Hemispheric Brain Networks in Major Depressive Disorder.” *Translational Psychiatry* 9(1):136.

Kessler, R. C., C. B. Nelson, K. A. McGonagle, J. Liu, M. Swartz, and D. G. Blazer. 1996. “Comorbidity of DSM-III-R Major Depressive Disorder in the General Population: Results from the US National Comorbidity Survey.” *The British Journal of Psychiatry. Supplement* (30):17–30.

Kim, Dongjae, Geon Yeong Park, John P. O′Doherty, and Sang Wan Lee. 2019. “Task Complexity Interacts with State-Space Uncertainty in the Arbitration between Model-Based and Model-Free Learning.” *Nature Communications* 10(1):5738.

Kumar, P., G. Waiter, T. Ahearn, M. Milders, I. Reid, and J. D. Steele. 2008. “Abnormal Temporal Difference Reward-Learning Signals in Major Depression.” *Brain : A Journal of Neurology* 131(Pt 8):2084–93.

Kumar, Poornima, Franziska Goer, Laura Murray, Daniel G. Dillon, Miranda L. Beltzer, Andrew L. Cohen, Nancy H. Brooks, and Diego A. Pizzagalli. 2018. “Impaired Reward Prediction Error Encoding and Striatal-Midbrain Connectivity in Depression.” *Neuropsychopharmacology* 43(7):1581–88.

Kunisato, Yoshihiko, Yasumasa Okamoto, Kazutaka Ueda, Keiichi Onoda, Go Okada, Shinpei Yoshimura, Shin-ichi Suzuki, Kazuyuki Samejima, and Shigeto Yamawaki. 2012. “Effects of Depression on Reward-Based Decision Making and Variability of Action in Probabilistic Learning.” *Journal of Behavior Therapy and Experimental Psychiatry* 43(4):1088–94.

LaFreniere, Lucas S. and Michelle G. Newman. 2018. “Probabilistic Learning by Positive and Negative Reinforcement in Generalized Anxiety Disorder.” *Clinical Psychological Science* 7(3):502–15.

Lee, Sang Wan, Shinsuke Shimojo, and John P. O’Doherty. 2014. “Neural Computations Underlying Arbitration between Model-Based and Model-Free Learning.” *Neuron* 81(3):687–99.

Lewinsohn, P. M., J. R. Seeley, R. E. Roberts, and N. B. Allen. 1997. “Center for Epidemiologic Studies Depression Scale (CES-D) as a Screening Instrument for Depression among Community-Residing Older Adults.” *Psychology and Aging* 12(2):277–87.

Li, Mi, Hongpei Xu, and Shengfu Lu. 2018. “Neural Basis of Depression Related to a Dominant Right Hemisphere: A Resting-State FMRI Study” edited by L. Piccardi. *Behavioural Neurology* 2018:5024520.

Maddox, W. Todd, Bharath Chandrasekaran, Kirsten Smayda, Han-Gyol Yi, Seth Koslov, and Christopher G. Beevers. 2014. “Elevated Depressive Symptoms Enhance Reflexive but Not Reflective Auditory Category Learning.” *Cortex; a Journal Devoted to the Study of the Nervous System and Behavior* 58:186–98.

Markman, Keith and Audrey Miller. 2006. “Depression, Control, and Counterfactual Thinking: Functional for Whom?” *Journal of Social and Clinical Psychology* 25.

De Martino, Benedetto, Stephen M. Fleming, Neil Garrett, and Raymond J. Dolan. 2013. “Confidence in Value-Based Choice.” *Nature Neuroscience* 16(1):105–10.

Miller, Kevin J., Matthew M. Botvinick, and Carlos D. Brody. 2017. “Dorsal Hippocampus Contributes to Model-Based Planning.” *Nature Neuroscience* 20(9):1269–76.

Miyagi, Madoka, Makoto Miyatani, and Takashi Nakao. 2017. “Relation between Choice-Induced Preference Change and Depression.” *PloS One* 12(6):e0180041–e0180041.

Ogishima, Hiroyoshi, Shunta Maeda, Yuki Tanaka, and Hironori Shimada. 2020. “Effects of Depressive Symptoms, Feelings, and Interoception on Reward-Based Decision-Making: Investigation Using Reinforcement Learning Model.” *Brain Sciences* 10:508.

Perez, Hector. 2014. *Brain Lateralization on the Emotional Processing of Clinically Diagnosed Anxious and Depressed Male Patients*.

Prescott, C. A., J. J. McArdle, E. S. Hishinuma, R. C. Johnson, R. H. Miyamoto, N. N. Andrade, J. L. Edman, G. K. Jr Makini, L. B. Nahulu, N. Y. Yuen, and B. S. Carlton. 1998. “Prediction of Major Depression and Dysthymia from CES-D Scores among Ethnic Minority Adolescents.” *Journal of the American Academy of Child and Adolescent Psychiatry* 37(5):495–503.

Quelhas, Ana Cristina, Mick J. Power, Csongor Juhos, and Jorge Senos. 2008. “Counterfactual Thinking and Functional Differences in Depression.” *Clinical Psychology & Psychotherapy* 15(5):352–65.

Rothkirch, Marcus, Jonas Tonn, Stephan Kohler, and Philipp Sterzer. 2017. “Neural Mechanisms of Reinforcement Learning in Unmedicated Patients with Major Depressive Disorder.” *Brain : A Journal of Neurology* 140(4):1147–57.

Sartorius, N., T. B. Ustün, Y. Lecrubier, and H. U. Wittchen. 1996. “Depression Comorbid with Anxiety: Results from the WHO Study on Psychological Disorders in Primary Health Care.” *The British Journal of Psychiatry. Supplement* (30):38–43.

Ubl, Bettina, Christine Kuehner, Peter Kirsch, Michaela Ruttorf, Carsten Diener, and Herta Flor. 2015. “Altered Neural Reward and Loss Processing and Prediction Error Signalling in Depression.” *Social Cognitive and Affective Neuroscience* 10(8):1102–12.

Weissman, M. M., D. Sholomskas, M. Pottenger, B. A. Prusoff, and B. Z. Locke. 1977. “Assessing Depressive Symptoms in Five Psychiatric Populations: A Validation Study.” *American Journal of Epidemiology* 106(3):203–14.

Zhang, Yuying, Rose Z. W. Ting, Marco H. B. Lam, Siu-Ping Lam, Roseanne O. Yeung, Hairong Nan, Risa Ozaki, Andrea O. Y. Luk, Alice P. S. Kong, Yun-Kwok Wing, Norman Sartorius, and Juliana C. N. Chan. 2015. “Measuring Depression with CES-D in Chinese Patients with Type 2 Diabetes: The Validity and Its Comparison to PHQ-9.” *BMC Psychiatry* 15(1):198.
