# Supplementary material for: Effects of subclinical depression on prefrontal–striatal model-based and model-free learning
Source: PLoS Comput Biol. 2021 May 14;17(5):e1009003. doi: 10.1371/journal.pcbi.1009003 (PMC8153417; doi:10.1371/journal.pcbi.1009003)
Supplement: Attachment — Submitted filename: Response to Reviewers_second round_final.docx [file pcbi.1009003.s004.docx]

**Reviewer's comments**

**Reviewer #1**: I thank the authors for the comprehensive response to my comments. I appreciate the more nuanced label of 'subclinical depression'. The changes to the task figure are helpful and the revision of the text with signposting has made the results a lot clearer and easier to follow.
>> Once again, we appreciate the reviewer’s insightful comments about our work. We also believe that the updates would help readers understand the details clearly.

A few things I would recommend:
1. Author should use numeric numbers (e.g. N = 63, instead of 'sixty-nine') for the number of participants (both in results and methods) for better readability.
>> (Reply 1-1) Thank you for the suggestion. We have changed the expression of the number of participants (i.e. sixty-five → 65, sixty-three → 63, and twenty-eight → 28).

2. The discussion would read better if the last paragraph is a concise conclusion, instead of ending abruptly after a limitations paragraph.
>> (Reply 1-2) Thank you for the constructive comments. We have added a short paragraph providing a concise conclusion as the last paragraph of the Discussion session. We have also restructured the paragraph of the Discussion to improve the readability.

*“Our work combines computational model-based and machine learning approach to investigate the cognitive dysfunction in subclinical depression. We believe that our findings not only help us establish a rich connection between impaired decision making and subclinical traits of depression, but also raise the possibility for investigating cognitive impairments extending from a normal to subclinical psychiatric diseases.”*

Overall, I believe the authors have sufficiently addressed my concerns.

**Reviewer #2**: The authors have done a good job responding to my comments. I have no further comments.

>> We greatly appreciate the reviewer’s comments which helped us significantly improve our paper.

**Reviewer #3**: I congratulate the authors on their job in clarifying most of the issues raised. I am of the opinion that the paper is much improved. However, I am still missing some information in their responses.
>> Thank you for sharing valuable insights with us, which have made it possible for us to significantly improve the paper.

1) re. 3.1: The authors now explain how they split up the sample in two groups, but as far as I can tell only for the fMRI sample (n=28). I am wondering, however, how they split up the groups in the overall sample (n=63)?
>> (Reply 3-1) We have applied the same criterion when splitting the whole participants into two groups. To fully clarify this, we have added the description to the legend of Fig 4.

*“The healthy and subclinical depression group were defined by using the standard cutoff criteria of the CES-D score.”*

2) re. 3.3: “subjects with a lower depression score more likely to use…”? Shouldn’t that be "higher" depression score?
>> (Reply 3-2) We apologize for the typo. Yes, subjects with a higher depression score have the tendency to use a suboptimal decision making. We have corrected the sentence accordingly.

3) 3.5: The response given by the authors is not sufficient in my view. I was asking about how the three main model-free measures are related, i.e., what is the relationship between overall learning, model-based learning, and model-free learning, across the entire sample? This is not explained in 3.3, as stated by the authors.
>> (Reply 3-3) The task performance, choice optimality, and choice consistency was intended to quantify the overall learning performance, the degree of contribution of model-based learning to the performance, and that of model-free learning to the performance, respectively (For more detail, please refer (Reply 3-2) from our previous responses). In this sense, the choice optimality and the consistency are independent measures, whereas the task performance reflects both the choice optimality and consistency.

These three model-free behavioral measures help us gain an intuitive understanding of the engagement of model-free and model-based learning strategies because to maximize the amount of rewards, participants should combine both the model-based and model-free learning in a way to best accommodate to the changes of the environmental structure. Indeed, this behavioral analysis motivated us to explore the possible ways in which model-based and model-free learning guides choice behavior.

4) I advise the authors to show the changes they made in the manuscript also in the response letter to reviewers. As it is, I cannot infer the changes that have been made by the authors in response to 3.6, 3.8, 3.11, which makes it impossible for me to review the changes.

>> (Reply 3-4) To fully reflect this, we have indicated the changes we made in the manuscript and the response letter.

(i) For Reply 3-6 We have added the details of the models in the section ‘Computational models’ in the Method section.

*“In summary, we tested the following models: (1) Arbitration model proposed in (Lee et al., 2014) (Arb), (2) Arb model additionally assigning separate learning rates to model-based and model-free system (use α_MB_, α_MF_ instead of α, Arb_α_), (3)~(5) Arb_α_ model with additional assumption that the degree of exploitation is function of the model choice probability (Arb_α,τ1_, τ_1_ = f(P_MB_, P_MF_), f: logistics (*$\tau=c_{1}+\frac{c_{2} -c_{1}}{1+e^{(-c_{3}*\left( P_{MB}-c_{4} \right))}}$*) ; Arb_α,τ2_, τ2 = f(P_MB_, P_MF_), f: linear (τ=P_MB_*c); Arb_α,τ3_, τ3 = f(P_MB_, P_MF_), f: weighted linear (τ=P_MB_*τ_MB_+ (1-P_MB_)*τ_MF_)).”*

(ii) For Reply 3-8 We have updated the description of the small volume correction in the ‘Whole brain analyses’ in Method section.

*“For right ilPFC, we ran a 10 mm small-volume correction to evaluate the effect of depression on the arbitration control. A 10 mm radius sphere is based on the previous work which applies a 10 mm sphere criterion in the lateral PFC region (Fujiwara et al. 2009). The bilateral ilPFC coordinates were defined following the previous work (Lee, Shimojo, and O’Doherty 2014).”*

(iii) For Reply 3-11 To avoid any potential misinterpretation, we have changed the words implying causality to relations. In abstract, we have changed the term ‘disrupts arbitration control’ to ‘accompanies the disrupted arbitration control’. In Potential clinical applications and limitations section, we have changed the term ‘explain’ to ‘would give a clue’.

*“Critically, we found that it accompanies the disrupted arbitration control between model-based and model-free learning in the predominantly inferior lateral prefrontal cortex and frontopolar cortex.”*

*“First, our neural results would give a clue why brain stimulation techniques such as repetitive transcranial magnetic stimulation to the frontopoloar cortex or deep brain stimulation to the ventral striatum are effective in alleviating depressive symptoms”*
